# Towards shaping picosecond strain pulses via magnetostrictive transducers

**DOI:** 10.1016/j.pacs.2023.100463

**Published:** 2023-02-17

**Authors:** Maximilian Mattern, Jan-Etienne Pudell, Karine Dumesnil, Alexander von Reppert, Matias Bargheer

**Affiliations:** a Institut für Physik & Astronomie, Universität Potsdam, 14476 Potsdam, Germany; b European XFEL, 22869 Schenefeld, Germany; c Institut Jean Lamour (UMR CNRS 7198), Université Lorraine, 54000 Nancy, France; d Helmholtz Zentrum Berlin, 12489 Berlin, Germany

**Keywords:** Picosecond ultrasonics, Magnetostriction, Ultrafast x-ray diffraction, Ultrafast photoacoustics, Nanoscale heat transfer, Negative thermal expansion

## Abstract

Using time-resolved x-ray diffraction, we demonstrate the manipulation of the picosecond strain response of a metallic heterostructure consisting of a dysprosium (Dy) transducer and a niobium (Nb) detection layer by an external magnetic field. We utilize the first-order ferromagnetic–antiferromagnetic phase transition of the Dy layer, which provides an additional large contractive stress upon laser excitation compared to its zero-field response. This enhances the laser-induced contraction of the transducer and changes the shape of the picosecond strain pulses driven in Dy and detected within the buried Nb layer. Based on our experiment with rare-earth metals we discuss required properties for functional transducers, which may allow for novel field-control of the emitted picosecond strain pulses.

## 1 Introduction

The shape and timing of laser-generated picosecond strain pulses encodes a multitude of information on the initial light–matter interaction, subsequent energy transfer processes and the sample structure. Using picosecond ultrasonics as a probing tool has various application scenarios, as this non-invasive technique provides nanoscale depth resolution of buried interfaces and sample defects that can be utilized for large scale imaging of elastic properties [1], [2], [3], [4].

It is attractive to advance from observing material properties to controlling them. Previous experiments with high amplitude strain pulses have demonstrated that propagating lattice strain may transiently change the polarization in piezo- and ferroelectric specimen [5], [6], assist or even drive metal–insulator phase transitions [7], [8], manipulate energy levels and emission spectra in quantum wells [9], [10] and induce a coherent magnetization precession [11], [12], [13] where the potential for magnetization switching is explored [14], [15].

**Fig. 1:**
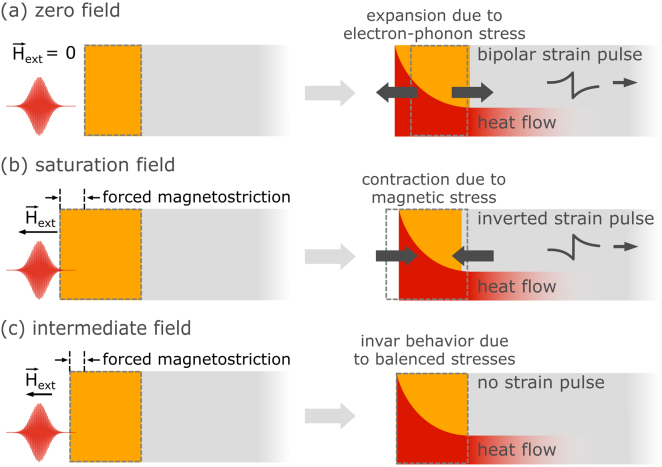
**Concept of field-controlled strain pulses:** A transducer exhibiting giant forced magnetostriction may ideally be tuned from an expansive zero field response (a) to a laser-induced contraction due to the release of a magnetic stress upon demagnetization (b) via an intermediate state of an ultrafast invar behavior (c).

The shape of the generated strain pulse is set by the sample geometry as well as optical, electronic and elastic properties of the transducer [16], [17]. Most experiments utilize a nanoscopically thin metal or semiconductor which expands upon laser-excitation due to electron and phonon stresses [18], resulting in a bipolar strain pulse with a leading compressive and a trailing tensile part [16], [17]. This shape can be tailored during sample growth by adding a non-absorbing capping to the transducer, which yields a unipolar pulse traveling towards the surface [19], [20], [21] or a train of pulses in case of a laser-excited superlattice [22], [23], [24]. The obtained strain amplitude is readily adjustable by tuning the absorbed pump pulse excitation energy density [25], [26] and multipulse excitation schemes can tailor involved strain pulse patterns [27], [28], [29], [30]. However, variations in the laser excitation parameters have only a limited effect on the overall shape of the strain pulse when the underlying mechanism for the stress generation remains unchanged. Controlling the sign and shape of the emitted strain pulses requires functional opto-mechanical transducers where the driving stress can be tuned from expansive to contractive within one device. This could yield inverted bipolar strain pulses and enable the emission of unipolar tensile pulses into adjacent capping layers. This concept based on a metallic transducer with giant forced magnetostriction i.e. a magnetic field dependent strain $\geq 10^{-3}$ is schematically depicted in Fig. 1. The zero field response is that of a normal metallic transducer (Fig. 1(a)), which could be overcome by the release of an additional field-induced stress upon laser-induced demagnetization as shown in Fig. 1(b). At an intermediate field where expansive electron–phonon stresses and a contractive magnetic stress are balanced one may even attain ultrafast invar behavior, which would allow for studying laser heating in absence of strain pulses as depicted in Fig. 1(c).

Previous works have demonstrated that magnetic stresses can arise on picosecond timescales in the context of laser-induced magnetic phase transitions [31], [32] and upon laser-induced demagnetization [33], [34], [35]. In rare earth metals the magnetic stress can even dominate over the electron–phonon stress contribution. It changes the strain response of the transducer from expansive to contractive upon cooling below the magnetic ordering temperature [36], [37], [38], [39]. The saturation of the magnetic stress contribution upon full demagnetization results in unconventional strain pulses due to a non-monotonic stress profile with a spatial sign change within an inhomogeneously excited transducer [36]. Ultrafast x-ray diffraction (UXRD) can be used to extract the rise time and the spatial distribution of the laser-induced stress by modeling the resulting strain in the transducer and buried detection layer [36], [39].

Here, we explore for the first time, how an external magnetic field can tune the picosecond strain pulses emitted by a rare earth transducer. Via UXRD, we observe this effect in a metallic heterostructure consisting of a dysprosium (Dy) transducer and a niobium (Nb) detection layer by utilizing the field-dependent first-order ferromagnetic–antiferromagnetic phase transition of Dy. This transition provides an additional contractive stress that rises with a timescale on the order of 10 ps if the Dy transducer is excited in a field-stabilized ferromagnetic phase. We identify an additional field-dependent stress, which is fast enough to modify the shape of the unconventional picosecond strain pulses derived from an antiferromagnetic Dy transducer [36] into the adjacent layers. This work opens a route towards field control of picosecond strain pulses via ultrafast forced magnetostriction.

In Section 2 of this article we introduce the quasi-static field- and temperature-dependent strain across the magnetic phase transition of the Dy layer. Section 3 contains the main experimental results on the field-dependent picosecond strain response within the transducer and the detection layer probed via UXRD. In Section 4 we summarize the results and discuss the desired transducer properties for advancing further towards full field-control of picosecond strain pulses.

## 2 Magnetostriction characterized by x-ray diffraction

The main ingredient for our functional Dy transducer is its giant magnetostriction that results in a negative thermal expansion at low temperatures. Magnetostriction on the order of 10^−3^ is common to the heavy rare earth elements Gadolinium through Erbium, which all exhibit an anomalous expansion along their hexagonal c-axis below their respective magnetic ordering temperature [40], [41]. We characterize the expansive magnetic strain of the Dy thin film using the temperature-dependent shift of the corresponding Bragg-peak.

**Fig. 2:**
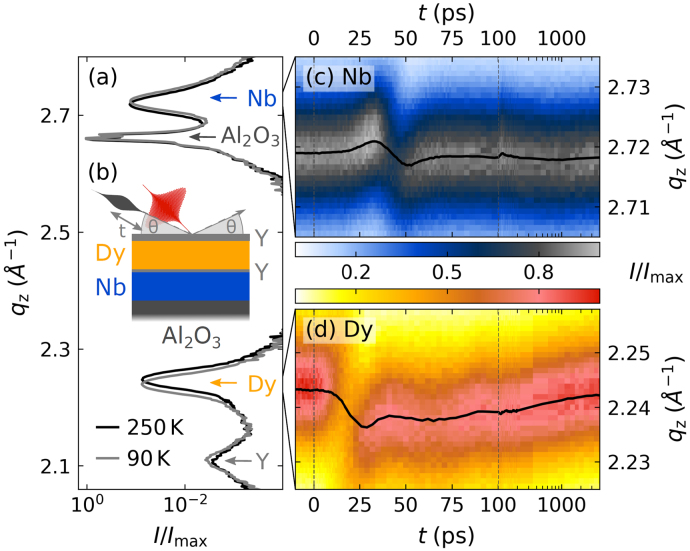
**Extraction of the lattice strain from a diffraction peak shift:** (a) x-ray diffraction intensity of our heterostructure under an applied in-plane field of 600mT as a function of the out-of-plane reciprocal space coordinate $q_{\text{z}}$ at two different temperatures. The diffraction curve exhibits four material-specific peaks that correspond to the average out-of-plane lattice constant of each constituent of our heterostructure, which has the layer sequence Y(20 nm)/Dy(80 nm)/Y(5 nm)/Nb(102 nm) on an ${\mathrm{Al}}_{2}O_{3}$ substrate. (b) Schematic depiction of the time-resolved pump-probe experiment where an x-ray pulse probes the evolution of the diffraction peaks for different times t after laser excitation. Panels (c) and (d) show the transient shift of the Nb and the Dy peak upon laser excitation with $7.2\;{\text{mJ/cm}}^{2}$ in the paramagnetic phase at 250 K.

Our sample consists of a layered metal heterostructure grown via molecular beam epitaxy on an ${\mathrm{Al}}_{2}O_{3}$ hcp-$\left(11\bar{2}0\right)$ substrate, as described previously [42], [43]. The sample structure is schematically depicted in Fig. 2(b). It contains a 102nm (110)-oriented Nb detection layer and an 80nm (0001)-oriented Dy transducer in between of two (0001)-oriented yttrium (Y) buffer layers (22nm on top of and 5nm below Dy). We study its near-equilibrium expansion via x-ray diffraction (XRD) by symmetric θ−2θ scans along the out-of-plane reciprocal coordinate $q_{\text{z}}$. A continuous wave x-ray tube combined with a Montel optic provides hard x-ray photons monochromatized to an energy of ≈8 keV, which have an extinction length on the order of micrometers into the metal stack. The x-ray diffraction intensity, shown in Fig. 2(a), thus exhibits material-specific Bragg peaks of all four materials within the structure. Their diffraction peak-positions are determined by the average out-of-plane lattice constant d of the respective material via $q_{\text{z}}=2\pi /d$. A comparison of the diffraction curves at 90K and 250K shows that the Dy layer exhibits a pronounced shift to larger $q_{\text{z}}$ indicating its contraction, while all other materials expand. Tracking the diffraction peak position during temperature and field variation yields the material-specific strain η as relative change of the lattice constant with respect to a reference state according to:

(1) $$\eta =\frac{d-d_{\text{ref}}}{d_{\text{ref}}}=\frac{q_{\text{z,ref}}-q_{\text{z}}}{q_{\text{z}}}\;.$$

**Fig. 3:**
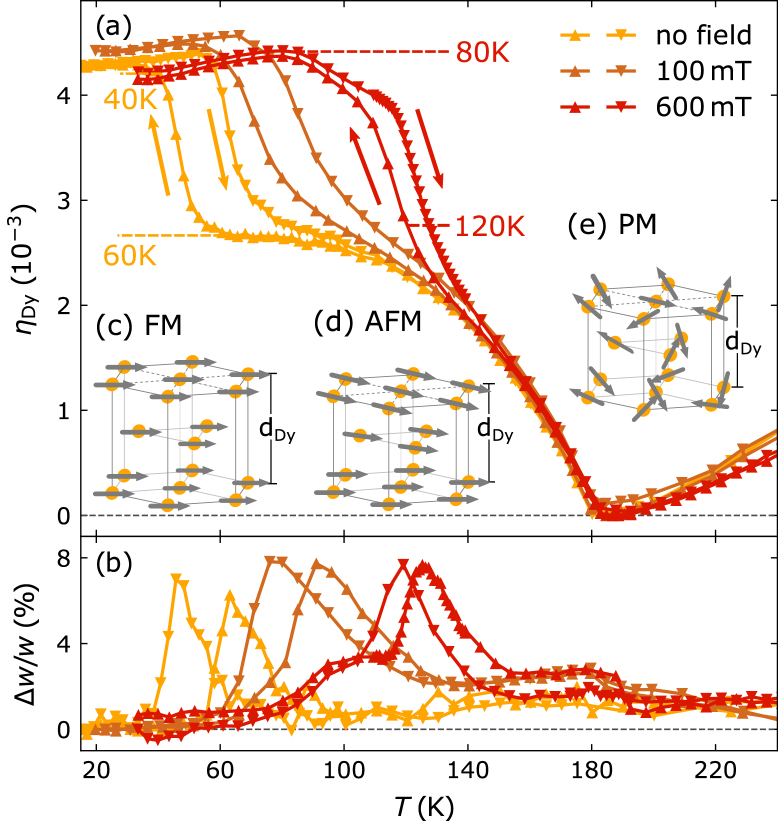
**Field-dependent strain in Dy at the FM-AFM transition:** (a) Temperature-dependent out-of-plane lattice strain of the Dy layer referenced to 180K for different in-plane B-fields. The symbols indicate the scan direction of the temperature (▴ for increasing and ▾ for decreasing). Panel (b) shows the corresponding change of the Bragg peak width that is increased through the FM-AFM phase transition due to a co-existence of both phases. Insets (c–e) schematically visualize the temperature-dependent type of magnetic order of the large magnetic moments localized on the Dy atoms.

The temperature-dependent out-of-plane lattice strain of the Dy layer along its hexagonal c-axis $\eta_{\text{Dy}}$ is depicted in Fig. 3(a). It is referenced to the minimum at 180K below which an anomalous expansion is observed that is concomitant to the onset of magnetic order. Bulk Dy hosts a helical spin order below $T_{\text{Néel}}=180\;\text{K}$ where the turn angle between the magnetic moments in the basal plane of the hcp structure decreases from 45° to 25° before it jumps to 0° at approximately 90K [44], [45], [46]. This first order phase transition from the antiferromagnetic helical order (AFM) to a ferromagnetic (FM) state is accompanied by an additional expansion along the out-of-plane direction. It is shifted to 40K in our thin film specimen due to growth induced strains [43], [47]. The insets Fig. 3(c–e) depict the order of the magnetic moments localized on the Dy atoms and the evolution of the out-of-plane lattice constant of Dy along its c-axis $d_{\text{Dy}}$ in the ferromagnetic, helical-antiferromagnetic and paramagnetic (PM) phase as function of temperature.

The unconventional magnetic order in rare-earth elements and magnetostriction arises from the oscillatory distance dependence of the RKKY exchange interaction. It couples the large but localized magnetic moments of the Dy 4f-electrons via a spin polarization of the delocalized 5d6s conduction band electrons. The resulting exchange-striction effect can be captured qualitatively by a simplified model for the Free energy per unit volume F for localized, interacting magnetic moments [40], [48], [49]:

(2) $$F=F_{\mathrm{elastic}}+F_{\mathrm{mag}}=\frac{1}{2}Y{\left(\frac{d_{\text{Dy}}-d_{\text{Dy,T}}}{d_{\text{Dy,T}}}\right)}^{2}-J\left(d_{\text{Dy}}\right)\;{\vec{M}}_{\text{A}}\cdot {\vec{M}}_{\text{B}}\;,$$

which merely consists of a harmonic potential with an elastic constant Y and a classical molecular field model for interacting sublattice magnetizations ${\vec{M}}_{\text{A}}$ and ${\vec{M}}_{\text{B}}$ that are coupled via a distance-dependent exchange constant $J\left(d_{\text{Dy}}\right)$. Here, ${\vec{M}}_{\text{A}}$ and ${\vec{M}}_{\text{B}}$ represent the sublattice magnetization of neighboring layers along the hexagonal c-axis of the Dy unit cell. Minimizing F after a Taylor expansion of $J\left(d_{\text{Dy}}\right)$ around the paramagnetic equilibrium lattice constant $d_{\text{Dy,T}}$ yields a magnetic strain:

(3) $$\eta_{\mathrm{mag}}=\left(\frac{d_{\text{Dy}}-d_{\text{Dy,T}}}{d_{\text{Dy,T}}}\right)=\frac{d_{\text{Dy,T}}}{Y}\frac{\partial J}{\partial d_{\text{Dy}}}M^{2}\cos\left(\phi \right)\;.$$

The magnetic strain is proportional to the square of the sublattice magnetization and the cosine of the average angle ϕ between neighboring magnetic moments. This simple expression for the exchange-striction put forward by Kittel [48] was already used in the first works by Darnell [40] as it suffices to rationalize the overall shape of the observed temperature dependent quasi-static strain depicted in Fig. 3(a). The appearance of the sublattice magnetization below $T_{\text{Néel}}=180\;\text{K}$ yields an expansive magnetic strain that superimposes the anharmonic phonon–phonon expansion that is visible in the PM phase. Lowering the temperature reduces the interlayer turn angle ϕ and increases the sublattice magnetization, which both enhance the magnetic strain contribution. A sudden increase in the magnetic strain contribution arises as ϕ changes step-like from ≈ $25^{\circ }$ to 0° during the first order magnetic phase transition from the AFM to FM phase. The accompanying magnetic strain observed in our thin film sample amounts to approximately $1.6\cdot 10^{-3}$. The first-order nature of the AFM-FM transition results in a hysteretic behavior of the strain, where the increased diffraction peak width (see Fig. 3(b)) indicates a coexistence of domains of both phases through the transition.

The application of an external in-plane magnetic field is known to stabilize the FM phase in Dy [50], [51], [52], which increases the first-order phase transition temperature. This is reflected in Fig. 3 by the shift of the hysteresis in the lattice expansion and the Bragg peak broadening to higher temperatures upon placing two large permanent NdFeB magnets next to the sample. The maximum attainable B-field in our experiment of ≈600 mT results in an increased out-of-plane lattice constant of Dy in the temperature region between 60K and 120K. In the following we utilize this field tunable expansive pre-strain within the FM-AFM phase transition to generate an additional magnetic stress upon laser-induced demagnetization and investigate its effect on the emitted strain pulses. A detailed discussion of the quasi-static magnetostriction in heavy rare earth elements is given elsewhere [47], [53], [54].

**Fig. 4:**
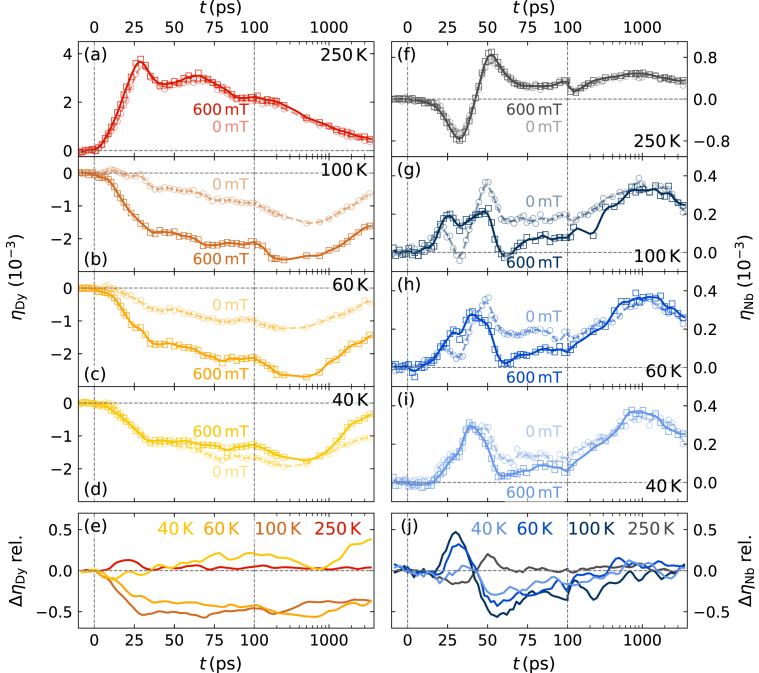
**B-field-dependent picosecond strain response:** Panels (a–d) show the laser-induced Dy strain with and without external in-plane field for the same excitation fluence^1^$F=7.2\;{\text{mJ/cm}}^{2}$ at different initial temperatures. Panels (f–i) depict the corresponding strain response of the Nb detection layer. The interpolated lines serve as guide to the eye. Panels (e) and (j) display the relative difference of the interpolated strain $\Delta \eta_{\text{rel}}=\left(\eta \left(600\;\text{mT}\right)-\eta \left(0\;\text{mT}\right)\right)/\max\left(\left|\eta \left(600\;\text{mT}\right)\right|\right)$ with and without applied external field for Dy and Nb, respectively.

## 3 Manipulation of picosecond strain pulses

Here, we discuss the laser-induced strain response of the Dy transducer and the Nb detection layer observed in a pump-probe UXRD experiment. The comparison of experiments with and without an applied in-plane magnetic field allows extracting the field-induced change of the picosecond strain pulse.

In the UXRD experiment, the heterostructure is excited by a 100fs-long p-polarized pump pulse with a central wavelength of 800nm that is incident under $\alpha_{\mathrm{in,laser}}\approx 38^{\circ }$ with respect to the sample surface. We probe the laser-induced change of the out-of-plane lattice constant by a 200fs hard x-ray pulse with a photon energy of ≈8 keV that is provided by a laser-driven table-top plasma x-ray source [55]. In the experiment we kept the incident angle θ of the x-ray pulse fixed at 16° and 20°. We track the position of the Bragg peaks of Dy and Nb using the reciprocal space slicing method [56]. The laser-induced peak shift (Fig. 2(c) and (d)) encodes the change of the mean out-of-plane lattice constant and provides the lattice strain following Eq. (1). The temperature- and field-dependent strain response of the Dy transducer and the Nb detection layer are depicted in Fig. 4(a–d) and (f–i), respectively. The following discussion only summarizes the main arguments to rationalize the observed picosecond strain response. A detailed discussion including a modeling of the driving stresses and the measured zero-field strain response have been published previously [36].

In the PM phase at 250K, we observe the conventional strain response of a laser excited metal. The Dy transducer response shown in (Fig. 4(a)) exhibits an expansion that reaches its maximum at ≈29ps, at the time when the expansive strain pulse front that starts at the sample surface has traversed the entire layer. The quasi-static expansion of Dy decays on a nanosecond timescale due to thermal energy transport to the adjacent layers. For $7.2\;\text{mJ}/{\text{cm}}^{2}$ excitation it amounts to $3\cdot 10^{-3}$ around the maximum, which corresponds to a temperature increase in the phonons of approximately 150K averaged over the inhomogeneously excited Dy layer that is thicker than the optical penetration depth of approximately 25nm. Partial back-reflections of the strain pulse at interfaces cause the damped oscillations that superimpose the strain from the quasi-static thermal expansion of the Dy layer. The expansion and the bipolar strain pulse with leading compression propagating through the heterostructure are driven both by electronic and phononic stress [18]. Due to the rapid electron–phonon coupling and similar Grüneisen parameters for electrons and phonons in rare earths a distinction of these two stress contributions has been unnecessary and generally only the combined expansive electron–phonon stress is distinguished from the contractive stress due to spin-disorder [36], [37], [38], [39], [57]. After the rapid electron–phonon coupling the phonons dominate the electron–phonon stress by far.

The evaluation of the corresponding Nb Bragg peak shift (Fig. 4(f)) yields the integral of the strain inside the detection layer. This separates strain pulses and thermal expansion in the time domain due to their different propagation speeds. The entrance of the leading compressive part of the bipolar strain pulse into Nb leads to a negative average strain of the detection layer. The exit of the compressive part and the entrance of expansive part of the strain pulse results in the subsequent expansion. The zero crossing in the Nb strain therefore marks the moment when the leading compressive and the trailing tensile part of the strain pulse have an equal amplitude within the detection layer. Heat diffusion from the Dy transducer into the Nb layer leads to the subsequent slow expansion trend, which peaks at approximately 1ns before it decays due to thermal transport towards the substrate. The strain response of the buried Nb detection layer thus provides direct access to the shape of the picosecond strain pulse.

The strain response observed in the AFM and FM phase changes qualitatively due to the presence of an additional laser-induced contractive magnetic stress contribution that arises below the magnetic order temperature $T_{\text{Néel}}=180\;\text{K}$. Already in the absence of an external magnetic field, the laser-induced reduction of the AFM order leads to a contractive stress in the Dy transducer which adds to the quasi-instantaneous expansive electron–phonon stress. The strain response of the unexcited Nb detection layer allows us to infer the resulting total stress profile since the strain pulse propagation translates the spatial stress profile to the time-domain. In Fig. 4(g–i) one finds that the Nb layer rapidly expands, although it is not optically excited. This contrasts the PM phase response and indicates that the contractive stress due to the spin disorder dominates in the Nb-near region of Dy. However, the corresponding field-free strain response of Dy shown in Fig. 4(b–d) displays a vanishing expansion within the first 10ps, which indicates that, on average, the expansive electron–phonon stress in Dy balances the contraction. We conclude that the Dy layer expands in the near-surface region and contracts near the Nb layer, which is supported by the composite shape of the strain response of the Nb detection layer. The detected strain pulse consists of a leading tensile part driven by the contraction of the transducer at the backside, which is followed by a conventional bipolar strain pulse launched at the expanding near-surface region. In the strongly excited near-surface region, the spin-disorder is already saturated but the electron–phonon system takes additional energy, which allows the expansive stress to dominate. These insights into the spatio-temporal stress contributions were confirmed by modeling the strain response of Dy and Nb in a previous publication [36] where we assume a saturable magnetic stress that rises on two timescales with a sub-picosecond contribution and a slower 15ps timescale that is necessary to rationalize the delayed slow rising contraction of Dy shown in Fig. 4(b–d). The relative amplitude of the saturable magnetic stress and the expansive electron–phonon stress depend on the initial sample temperature and excitation fluence, which affects the strain response of the Dy and Nb layer as displayed in Fig. 4. An extended overview of the temperature- and fluence-dependent strain response is provided in the supplementary material in Fig. S1 and S2.

The application of an external magnetic field now represents an additional degree of freedom for the manipulation of the picosecond strain response as it shifts the FM-AFM phase transition of Dy. Fig. 4 compares the strain response of Dy and Nb with and without external magnetic field under otherwise identical excitation conditions. The field-independent response in the PM phase at 250K and the field independent thermal expansion contribution in the Nb strain represent an experimental confirmation for the comparable energy deposition. The difference in the picosecond strain response therefore originates from a manipulation of the magnetic stress contribution by the external field. Fig. 4(e) and (j) display the time-dependent relative difference in the strain response of Dy and Nb for different initial sample temperatures. At 60K and 100K we observe an enhanced contraction of Dy by $1.2\cdot 10^{-3}$ that originates from the reduction of the field-activated magnetic stress and corresponds to an additional average contractive field-induced stress of 100MPa, which rises with a timescale on the order of 10 ps that is in line with the loss of the FM order parameter reported previously [58]. This additional contractive stress contribution is fast enough to change the shape of the emitted strain pulse compared to the zero-field response and yields an inverted bipolar difference signal in the strain of the Nb detection layer. The observed difference with and without field resembles the strain difference observed below and above the transition temperature $T_{\text{C}}$, which is displayed in Fig. S3 of the supplementary material. This confirms that the additional field-activated stress contribution indeed originates from the FM-AFM phase transition. The field-stabilized FM-phase exhibits an angle ϕ≈0 between the neighboring magnetic moments, which maximizes the magnetic pre-strain (see Eq. (3)) that can be released upon laser excitation. This additional contractive stress is only induced if Dy is driven across the FM-AFM phase transition which we estimate to occur on average at each initial sample temperature for $7.2\;{\text{mJ/cm}}^{2}$ based on the temperature increase in the phonons at 250K. The first-order nature of the phase transition introduces a threshold fluence that is necessary to heat Dy above its FM-AFM transition temperature (120K). Therefore, below the threshold fluence the excitation does not induce the contractive stress of the FM-AFM phase transition and the Dy transducer expands upon laser excitation as demonstrated by a fluence dependent study shown in Fig. S2 of the supplementary. At 40 K Dy is in its FM state regardless of the external field and thus the strain difference vanishes. We observe a field-dependent change of the picosecond strain pulse in the temperature range between 60K and 120K where the applied magnetic field stabilizes the FM order in the otherwise AFM phase (Fig. 3). The overall shape of the emitted strain pulse thus depends on the excitation fluence, sample temperature and external field as elaborated in the supplementary material.

The manipulation of picosecond strain pulses by a field-tunable, contractive magnetic stress originating from an excitation of the magnetic order is limited by the respective stress rise time in comparison to the subpicosecond rise of the expansive electron–phonon stress. The laser-induced loss of the magnetic order parameter in the FM-phase of Dy has been found to occur with a (6±2)  ps time constant [58]. This is relatively slow in comparison to the (sub)-picosecond rise of the electron–phonon stress [36]. In contrast, it is interesting to note that the ferromagnetic rare-earth element Tb has been reported to exhibit a subpicosecond demagnetization [59], [60] and quasi-static measurements indicate a similarly strong magnetostriction [41]. The combination of ultrafast demagnetization and giant magnetostriction renders it to be a suitable candidate for the manipulation of picosecond strain pulses with high-frequency components in the THz range. However, advanced models are necessary to establish a direct relation between demagnetization measurements and the magnetic stress generation. This is especially true for rare earth elements which exhibit large but localized magnetic moments hosted by 4f-electrons that are coupled indirectly via the 5d6s conduction band electrons.

## 4 Conclusion

In summary, this work demonstrates the manipulation of picosecond strain pulses emitted by a rare-earth transducer, which is either in its AFM or FM state depending on the applied external B-field. We find that the quasi-static magnetic strain between these two states of $1.6\cdot 10^{-3}$ can be released upon laser-excitation creating an additional contractive stress that rises within tens of picoseconds. This additional contractive stress adds an inverted bipolar strain pulse to the already unconventional picosecond strain launched by an inhomogeneously excited Dy transducer into a buried detection layer [36].

Based on this study we identify the following material properties of a transducer layer that are desired for realizing a field control of picosecond strain pulses via magnetostriction as initially proposed in Fig. 1: (1) Subpicosecond demagnetization for a complete compensation of the fast rising electron–phonon stresses that are inherent to laser-excited metals. (2) Giant forced magnetostriction with magnetic strains on the order of 10^−3^ that dominates over the expansive electron–phonon stress. (3) Nanoscopic films so that a nearly homogeneous laser-excitation of the transducer yields a constant sign of the stress within functional transducer. (4) Room-temperature magnetization would extend the applicability to non-cryogenic surroundings. (5) Flexible growth by sputtering methods with little requirements on the capping layers will aid device design.

Interesting material systems in that regard are Galfenol (FeGa) [61], Samfenol (SmFe) [62] and in particular Terfenol-D (Tb${}_{x}$Dy${}_{1-x}$Fe${}_{2}$ with x≈0.3) [63], which can be grown epitaxially on (110) oriented Nb [64]. Although these materials often occur as amorphous alloys it is possible to track the shape of the emitted strain pulses via UXRD in a crystalline detection layer adjacent to the transducer. The realization of field tunable strain pulses will be an important step towards controlling the strain driven responses of buried functional layers on picosecond timescales. This may allow for studies of ultrafast heating in absence of strain pulses, which are hitherto inseparable.

## Declaration of Competing Interest

The authors declare that they have no known competing financial interests or personal relationships that could have appeared to influence the work reported in this paper.

## Data Availability

Data will be made available on request.

## Appendix A Supplementary data

MMC S1

## Footnotes

1 We report the fluence F utilizing top-hat approximation that distributes the incident pump-pulse energy E homogeneously across an area A represented by an ellipse with the major axes ($d_{x},d_{y}$) which are determined from the corresponding 1/e values of the 2D Gaussian fit to the laser intensity profile. It is calculated according to $F=E/A=4E\sin\left(\alpha_{\mathrm{in,laser}}\right)/\left(\pi d_{x}d_{y}\right)$.
