# Supplementary material for: Towards shaping picosecond strain pulses via magnetostrictive transducers
Source: Photoacoustics. 2023 Feb 17;30:100463. doi: 10.1016/j.pacs.2023.100463 (PMC9982602; doi:10.1016/j.pacs.2023.100463)
Supplement: MMC S1 [file mmc1.pdf]

Supplementary material to:
Towards shaping picosecond strain pulses via magnetostrictive transducers

Maximilian Mattern[a], Jan-Etienne Pudell[a,b], Karine Dumesnil[c], Alexander von Reppert[a], Matias Bargheer[a,d]

[a] *Institut für Physik & Astronomie, Universität Potsdam, 14476 Potsdam, Germany*
[b] *European XFEL, 22869 Schenefeld, Germany*
[c] *Institut Jean Lamour (UMR CNRS 7198), Université Lorraine, 54000 Nancy, France*
[d] *Helmholtz Zentrum Berlin, 12489 Berlin, Germany*

In this supplementary we provide a systematic overview of the picosecond strain response of the Dy transducer and the Nb detection layer as function of the initial sample temperature and the fluence. Furthermore, we compare the strain response at temperatures below and above the ferromagnetic order temperature T_C to extract the additional stress that arises by exciting the Dy transducer in its ferromagnetic (FM) phase in comparison to an excitation in the antiferromagnetic phase (AFM) above T_C.

Fig. S1 displays the temperature-dependent strain response of the Dy and the Nb layer without (panels (a) and (b)) and with external magnetic field (panels (c) and (d)) for a relatively high laser excitation fluence of

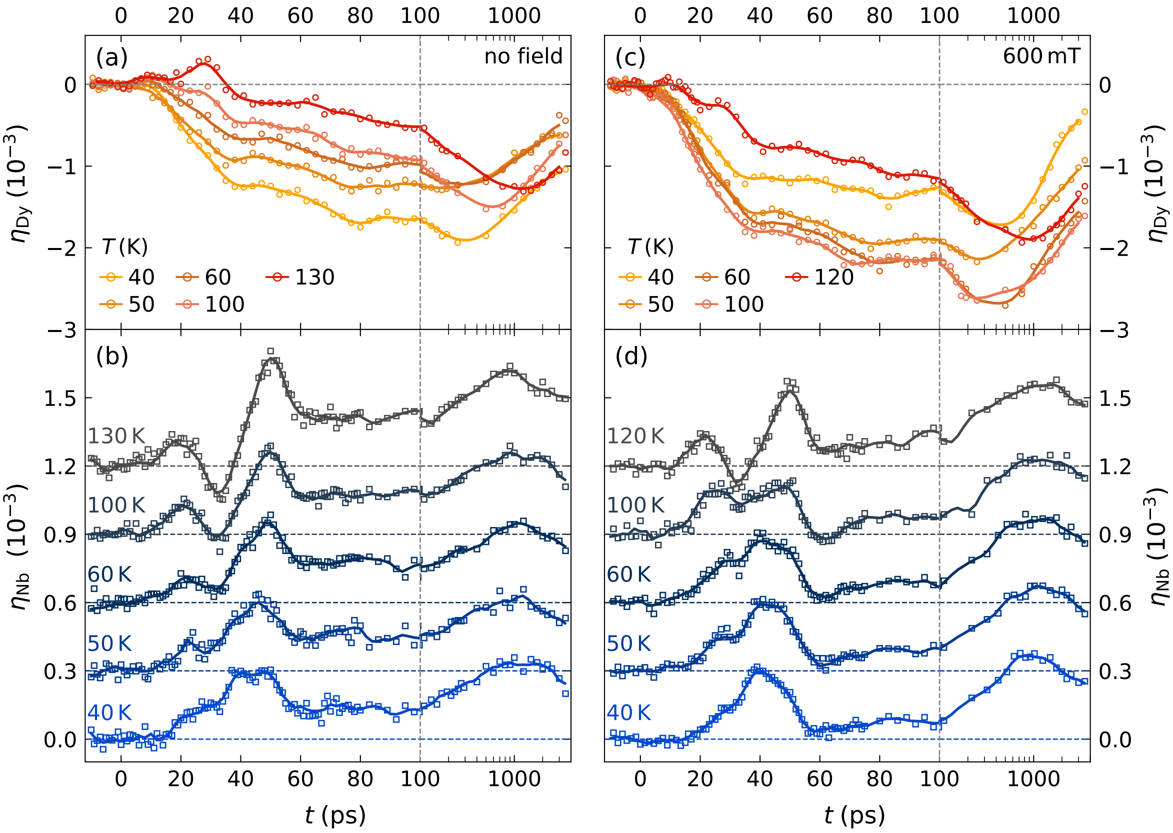

Figure S1: **Side by side comparison of the temperature-dependent strain response with- and without an external field:** The strain response of (a) the Dy transducer and (b) the Nb detection layer for $\approx 7.2\,\mathrm{mJ/cm^2}$. Panels (c) and (d) provide the Dy and Nb strain response for comparable excitation conditions under the application of an in-plane B-field of 600 mT. The interpolated solid lines serve as a guide to the eye.

7.2 mJ/cm^2. This depiction facilitates the quantitative comparison of the temperature-dependent contraction of the Dy transducer and the driven unconventional picosecond strain pulse detected in Nb. The resulting composite shape of the strain response is the superposition of a bipolar strain pulse driven by an expansive total stress at the front side of the transducer and an expansive strain pulse driven by a contraction of Dy at the backside. This complex stress profile within the inhomogeneously excited Dy transducer originates from a saturation of the contractive magnetic stress contribution upon full demagnetization at the front side of the layer where the expansive electron-phonon stress dominates the contraction for high fluence. Independent of the external field, the composite shape of the strain response of the Nb layer becomes more pronounced with increasing sample temperature, since a larger fraction of the Dy transducer is fully demagnetized and the expansive electron-phonon stress prevails. In the case of an applied external magnetic field, the composite shape of the strain response of Nb is less pronounced, which indicates the presence of an additional laser-induced contractive stress that counterbalances the expansive electron-phonon stress if Dy is excited in the FM phase. In addition, the external field influences the temperature dependence of the contraction of the Dy transducer. While in the zero-field case the maximum contraction is attained for a sample temperature of 40 K, the maximum contraction within an external field occurs at 100 K. This is related to the increase of the transition temperature from 40 K to around 120 K by the external field. Therefore, the maximum contraction of Dy occurs in both cases at initial sample temperature slightly below the respective transition temperature.

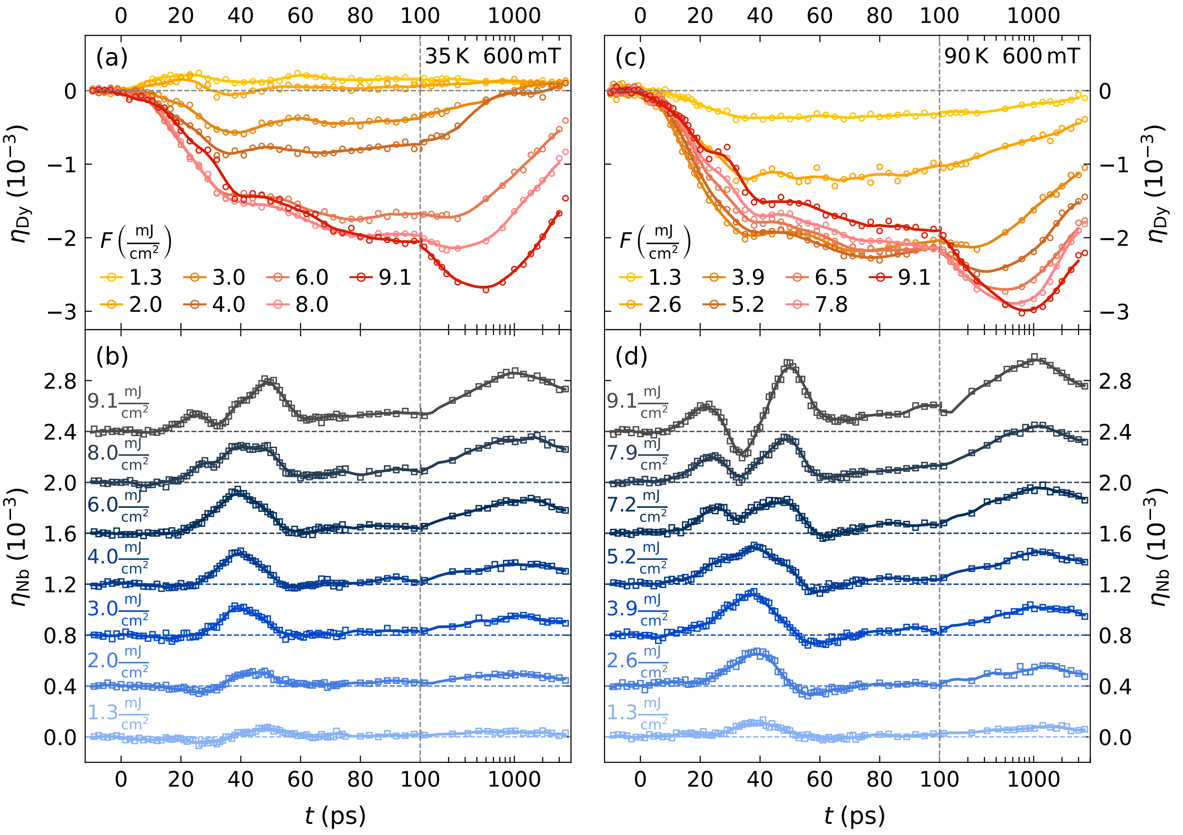

Figure S2: **Fluence-dependent strain response at temperatures below T C:** The excitation fluence-dependent strain response η of the Dy transducer (a) and the Nb detection layer (b) at T = 35 K for an in-plane B-field of 600 mT. The fluence-dependent strain reponse at a fixed start temperature T = 90 K is depicted in panels (c) and (d). The interpolated solid lines serve as a guide to the eye.

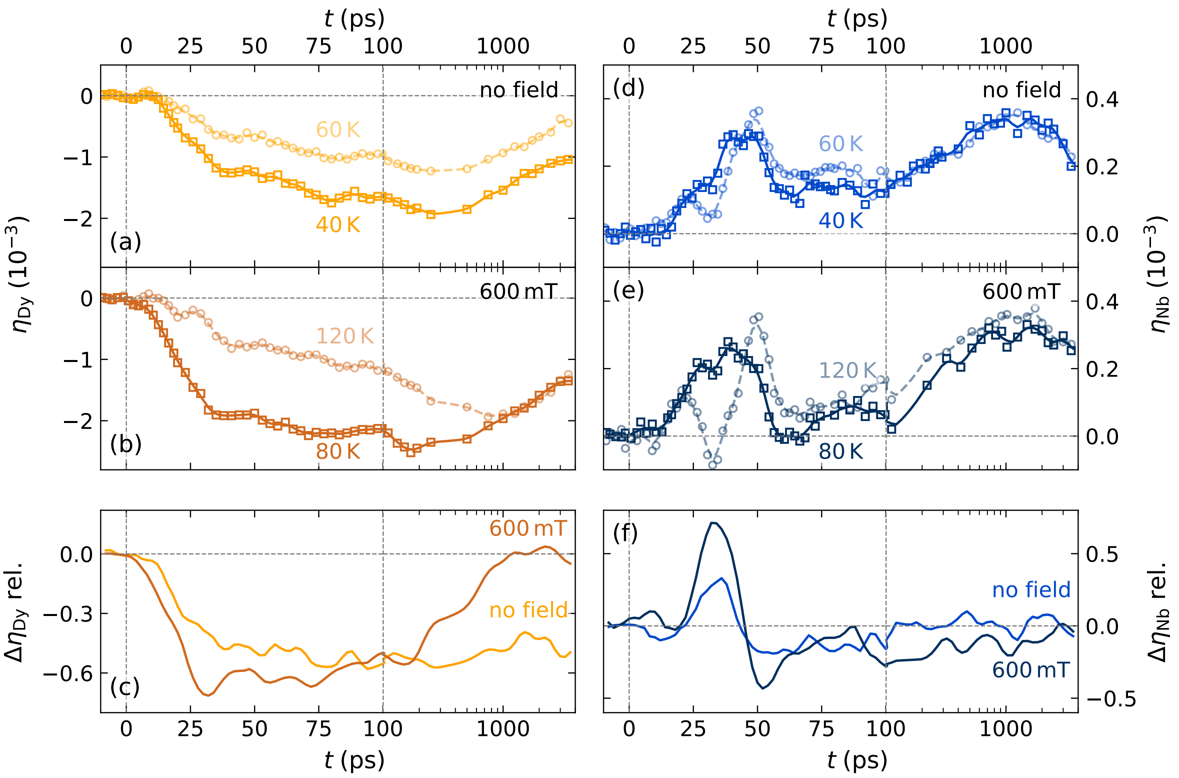

Figure S3: **Extraction of strain signature of the FM-AFM phase transition of Dy:** Panels (a) and (d) display the field free strain response above (60 K) and below (40 K) the transition temperature T_C of Dy and Nb for the same incident fluence of 7.2 mJ/cm^2. Panels (b) and (e) display the corresponding strain response within an external magnetic field of 600 mT below (80 K) and above (120 K) above the field-elevated transition temperature. The interpolated lines serve as guide to the eye. Panels (c) and (f) display the relative difference $\Delta\eta_{\text{rel}} = (\eta(\text{belowT}_\text{C}) - \eta(\text{aboveT}_\text{C})/\max(|\eta(\text{belowT}_\text{C})|)$ of the interpolated strain response at temperatures

above and below the FM-AFM transition temperature in the absence of a magnetic field and for 600 mT, which provides access to the additional laser-induced stress arising from exciting Dy in its FM phase.

The variation of the excitation fluence furthermore affects the strain response of the Dy transducer in a similar way as the effect of the inital sample temperature. Figure S2 displays the fluence-dependent strain response of the Dy and the Nb layer within an external magnetic field starting in the FM phase of Dy at 35 K well below the transition temperature (panels (a) and (b)) and at 90 K (panels (c) and (d)) only slightly below the transition temperature. In the high fluence regime we observe a saturation of the contraction of Dy for both start temperatures. At 90 K the contraction of Dy even decreases with increasing fluence. This saturation indicates a complete loss of the magnetic order within the laser-excited transducer region, which is reflected by the appearance of the composite shape in the Niobium strain. It is interesting to note that for experiments at 35 K, i.e. well below the transition temperature, Dy expands for low fluences which drives a conventional bipolar strain pulse as seen in the Nb strain response. However, at higher fluences or at a higher initial sample temperature only slightly below the transition temperature at 90 K Dy contracts leading to a unipolarly shaped strain response of Nb. This indicates that overcoming a threshold fluence is necessary to release the expansive magnetic pre-stress in the FM phase, whereas in the helical AFM phase no threshold behavior is observed. From the strain response of Dy at 250 K we estimate the average phonon temperature increase to 150 K for an excitation of 7.2 mJ/cm^2. We have confirmed that the laser-induced strain and thus the phonon temperature increase in the PM phase scales linearly with the excitation fluence (not shown). Below the magnetic order temperature T_N = 180 K the excitation of magnetic degrees of freedom reduces the laser-induced increase in the phonon temperature. In contrast, the

heat capacity decreases at low temperature which increases the overall temperature increase. Considering these effects, we estimate the average temperature increase for 2 mJ/cm^2 at 35 K to approximately 60 K. Therefore, Dy is not heated above the transition temperature of 120 K for an applied field. The absence of an induced contractive stress from the FM-AFM phase transition results in the observed expansion of Dy. In contrast, at 90 K the average temperature increase is sufficient to heat Dy above T_C inducing the phase transition its contractive stress dominates the expansive electron-phonon stress that results in a contraction of the Dy layer.

In order to extract the influence of the FM-AFM phase transition of Dy on the strain response of Dy and Nb we compare experimental results at temperatures below and above the field-dependent transition temperature T_C in Fig. S3. Panels (a) and (d) display the field free strain response with a transition temperature of 60 K and panels (b) and (e) the strain response within an external magnetic field of 600 mT increasing the transition temperature to 120 K. Independent of the external magnetic field, we find a larger contraction of the Dy transducer and a more unipolar shaped strain response of the Nb layer below the respective transition temperature. The corresponding strain difference depicted in panels (c) and (f) shows that the influence of the additional magnetic stress from starting in the FM phaseresembles the field-dependent difference in Fig. 4 of the main text. It rises within the first tens of picoseconds and results in an inverted bipolar strain wave that superimposes the unconventional strain wave in the Nb layer. This supports the interpretation in the main text that the additional field-induced stress originates from enabling the stress associated with the FM-AFM phase transition by stabilizing the FM phase before laser-excitation. However, the type of experiment presented here does not only contain the contribution of the phase transition but also involves different start temperatures affecting the relative difference. This becomes relevant in the case of the field-elevated transition temperature, since the large temperature difference between below and above T_C and their proximity to the complete loss of the magnetic order at $T_N = 180$ K additionally affects the saturation of the magnetic stress, which adds to the change induced by the additional expansive stress released in the FM phase. This enhances the additional contraction of Dy and the additional inverted bipolar strain pulse contribution in Nb at 80 K with respect to 120 K in comparison to the strain difference between 40 K and 60 K. Furthermore, the field-induced broadening of the FM-AFM phase transition causes a faster recovery of the FM phase by cooling to the substrate at 80 K than at 40 K in the absence of an external magnetic field. This results in a vanishing strain difference in Dy after 1 ns for 600 mT while the FM stress stays released up to several nanoseconds causing a long-lasting difference in the strain response of Dy without an applied magnetic field.
